# A Comparison of Comprehension Processes in Sign Language Interpreter Videos with or without Captions

**DOI:** 10.1371/journal.pone.0127577

**Published:** 2015-05-26

**Authors:** Matjaž Debevc, Danijela Milošević, Ines Kožuh

**Affiliations:** 1 Institute of Media Communications, Faculty of Electrical Engineering and Computer Science, University of Maribor, Maribor, Slovenia; 2 Department for Information Technology, Faculty of Technical Sciences Čačak, University of Kragujevac, Čačak, Serbia; Beijing Normal University, CHINA

## Abstract

One important theme in captioning is whether the implementation of captions in individual sign language interpreter videos can positively affect viewers’ comprehension when compared with sign language interpreter videos without captions. In our study, an experiment was conducted using four video clips with information about everyday events. Fifty-one deaf and hard of hearing sign language users alternately watched the sign language interpreter videos with, and without, captions. Afterwards, they answered ten questions. The results showed that the presence of captions positively affected their rates of comprehension, which increased by 24% among deaf viewers and 42% among hard of hearing viewers. The most obvious differences in comprehension between watching sign language interpreter videos with and without captions were found for the subjects of hiking and culture, where comprehension was higher when captions were used. The results led to suggestions for the consistent use of captions in sign language interpreter videos in various media.

## Introduction

With the rapid development of high-speed data transmission and video compression techniques the use of sign language interpreter videos has evolved. The deaf and hard of hearing have started using it to a greater extent when obtaining information from television programmes, films and web sites. Thus, sign language interpreter videos are for the deaf and hard-of-hearing who use sign language an important tool for interpreting spoken and/or written words [1–4].

Sign language refers to a visual–gestural language that differs from spoken/written language. It has its own morphological, syntactic, and grammatical rules and is considered to be less strict in word order than some spoken/written languages [5–6]. The ability to use sign language extensively depends on the users’ experiences and their exposure to sign language [1,7]. It is usually considered to be the primary language of many deaf people and those who lost their hearing before developing spoken language consider written/spoken language to be a second language. What is more, increased sign language ability also supports written sentence comprehension at the levels of individual words and syntax [8].

**Fig 1:**
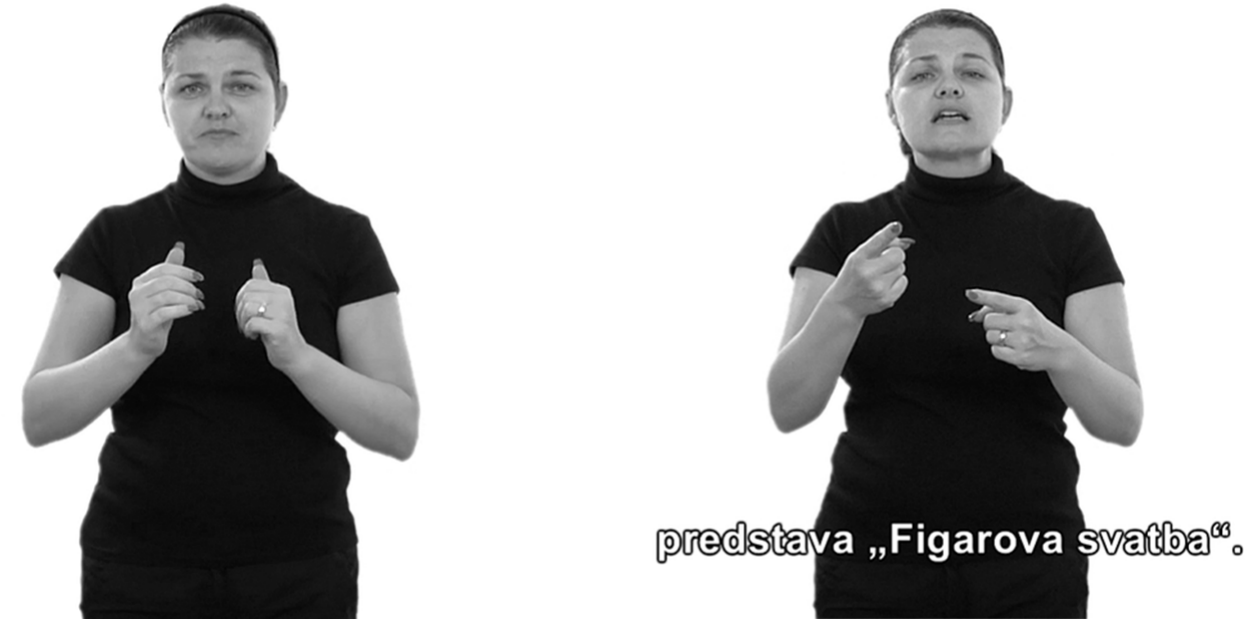
Two examples of the use of sign language interpreter videos on a website. (a) Sign language interpreter video without captions. (b) Sign language interpreter video with captions.

Subsequently, when these persons are exposed to written words, they often experience a degree of frustration as they encounter difficulties with the comprehension of more advanced texts. Therefore, the inclusion of captions into a sign language interpreter video could be a solution. Fig 1 shows examples of two different types of sign language interpreter videos that are commonly used worldwide. The video on the left provides information in sign language only, whereas the video on the right provides information both in sign language and captions. The most obvious difference between the videos is that when viewing the video on the left, viewers obtain information in sign language only, whereas when viewing the video on the right they can concurrently obtain information from two sources: signing and captions. The individual mentioned in this manuscript has given written informed consent (as outlined in PLOS consent form) to publish.

In existing research, there have been many attempts to address the benefits of captioning for deaf and hard of hearing sign language users. First of all, several studies have examined the effects of using captions in non-signing content on deaf and hard of hearing viewers’ comprehension. In particular, it was substantiated that accessing information in television programmes and films without captions is difficult for deaf and/or hard of hearing viewers [9–14], whereas the inclusion of captions either improves deaf and/or hard of hearing viewers’ comprehension [11, 15] or not [12, 16]. Additionally, previous studies also examined the use of different styles of captions [17] and caption speed [18, 19] in television programmes. They found no significant differences in deaf and hard of hearing viewers’ comprehension scores when using verbatim, standard, and edited captions [17]. However, it was found that more proficient deaf and hard of hearing readers comprehend captions better than less proficient readers, whereas the speed of captions also influences viewers’ comprehension [17, 18]. For instance, De Linde & Kay [19] confirmed that as many as 84% of deaf school children were not able to read at the rate of speech on television. A few studies have even gone one step further and recorded the eye movements of viewers watching captioned videos, as well as analysed the reading process for captions [20–22].

Secondly, only a few studies have investigated the effect of using captions in signing content. It was found that the presence of captions in video clips together with a sign language interpreter has a positive effect on deaf learners’ understanding of the content of educational material [23,24]. In line with that, the Federal Communications Commission (FCC) will require captioning on video clips from TV starting in 2016, and that is reported as the great progress of National Association for the Deaf [25]. The European Federation of Hard of Hearing People (EFHOH) has a vision of full inclusion for people with hearing loss with regard to access to media. Their report, entitled “State of subtitling access in EU 2011,” calls on EU Member States to respect the rights of deaf and hard of hearing citizens to have full access to media via subtitling by 2020 [26]. It derives from the EU Disability Strategy [27,28] and the UN Convention on the Rights of Persons with Disabilities [29]. Accordingly, it is advantageous that previous studies have extensively examined the effect of using captions on deaf and hard of hearing viewers’ comprehension in television programmes and films from different perspectives. However, there is a lack of research on the effects of using captions in sign language interpreter videos, where the themes of content displayed in these videos are also considered.

In this regard, the main aim of the current study was to find out what the increment in comprehension of the content of sign language interpreter videos was, when captions were included. To be more specific, we compared the comprehension scores of sign language interpreter videos with or without captions. Additionally, we aimed to investigate the differences in comprehension scores between different topics (sport, hiking, shopping, and culture) presented in sign language interpreter videos with, or without, captions. While the study did not determine the accuracy of the sign language interpreters, it has been approved by the Ethics Commission at ETH Zürich, Switzerland. Abovementioned aims of the study were achieved, since the paper presents positive effects of captions on deaf and hard of hearing viewers’ comprehension of sign language interpreter videos.

The paper is organized as follows. We start by providing background for the paper, where the need for a sign language interpretation video and captioning are presented. Then we describe the experiment, where experimental design, the pre-experiment, and main experiment are presented. A discussion and conclusion follow, where the findings are described and recommendations for future work are outlined.

## Background and Literature Review

### The need for sign language video interpretation

Deaf persons who were born deaf and have never heard a sound may have great difficulties in learning to speak or read auditory–vocal languages. The fact that the average deaf high school graduate is only able to read at a lower level [30] demonstrates the difficulty a deaf person can experience in this area.

Difficulties with reading are also evident in website usage. Websites mainly provide content in written form, which is almost illegible for deaf users. A study conducted by Fajardo, Cañas, Salmerón, and Abascal [31] discussed some of the problems regarding web accessibility for deaf persons. Only a few websites provide information in sign language, mostly through embedded video files showing sign language speakers translating a written text into sign language. From these studies we can conclude that for deaf and hard of hearing people, it is necessary to use sign language interpretation both for communication between them and hearing people, as well as to capture written text from different media, including websites.

### Captions for deaf and hard of hearing individuals

In existing research, there have been several attempts to find the positive implications of caption use. A wide range of research studies reported positive usage of captions supporting deaf persons in the learning of scientific literature and enhancing their reading vocabulary and comprehension skills [32–33]. In addition, Jelinek Lewis and Jackson [11] found in their study an improvement in the perception of information for deaf persons, especially for deaf students with a higher level of reading ability, who achieved better results by perceiving captions. It has also been shown that deaf students learn more when they have learning materials presented in more than one modality [34], such as using graphics or video with closed captioning [35–37]. Accordingly, these studies revealed that simultaneous processing (audio, video, and captions) enhances learning.

Moreover, Yoon and Kim [23] examined the positive effects of caption usage on deaf students’ content comprehension, cognitive load, and motivation in online learning. It was found that deaf students experienced a significant advantage in comprehension when they simultaneously used sign language video and captions. They propose that captions should be provided along with sign language video clips and the quality of captions should be taken into consideration in terms of the literacy levels of the learners. If the text is too difficult to understand, students’ motivation will fall. The main difference between our study and their study is that they developed a system where captions are displayed separately from the sign language interpreter video, while we study the use of captions inside the sign language interpreter video.

Furthermore, Szarkowska et al. [17] found in their eye tracking study that viewer comprehension of a video clip with the standard vs. edited simplified captions was high, at approximately 70%. They found that standard captions do not cause cognitive dissonance both for the deaf (who often use lip reading) as well as for the hard of hearing, who often compare audio based dialogue and captions. On the contrary, when using edited slower captions, participants achieve lower comprehension, as their processing may be hampered by discrepancies between the dialogue and the caption text.

However, Gulliver and Ghinea [9] similarly investigated the impact of captions on deaf and hearing people’s ability to assimilate information when watching multimedia video clips, but found no significant effect. Thus, they concluded, ‘captions do not necessarily provide deaf users with a greater level of information from video, but that the information assimilated from captions provides a greater level of context of the video’ (p. 384).

Margaret and Dorothy [38] additionally found that the success of caption usage strongly correlates with the level of reading knowledge and that the ability to read is the most important prerequisite for using captions in learning. Students who are better at reading written material can also read captions more easily.

In addition to the aforementioned research studies, accessibility standards and guidelines have addressed the implications of caption use. For instance, the Web Content Accessibility Guidelines (WCAG) [39,7] published by the Web Accessibility Initiative (WAI) provide advice on accessibility for multimedia material, such as animation and video, which is very general and does not define whether captions should be included in a sign language interpreter video or not. The WCAG states that when it comes to the visual components of multimedia, deaf or hard of hearing persons need captions or sign language interpretation, but it does not mention where and how.

Similarly, more detailed requirements for signing and captioning are provided by the European Broadcasting Union (EBU) [40] on Access Services, which precisely defines recommendations for people with disabilities in the broadcasting industry. The report includes a thorough list of recommendations for improving the readability of captioning, and two possible approaches for deaf signing: the use of signed videos and synthetic virtual signing.

Therefore, it is still not clear in any guidelines or in any research study whether or how captions have to be included in the sign language interpreter video, which is the main aim of our study, as presented in the current paper. In light of the above-mentioned problems, we conducted an experiment aimed at substantiating the use of captions in sign language interpreter videos.

### Importance of Using Captions for Sign Language Users—the Experiment

To outline the benefit of using captions in sign language interpreter videos, we evaluated sign language users’ comprehension of the content presented in these videos. The main objective was to examine how using captions in a sign language interpreter video affected the viewers’ comprehension of the content.

Methodology for this evaluation scenario was to perform the following three steps evaluation: (1) experimental design, (2) pre-experiment, and (3) main experiment. Before the study began, the experiment was approved by the Ethics Commission at ETH Zürich, Switzerland, and all participants were asked to sign a written consent form to participate in the study. At the Ljubljana School for the Deaf, Ljubljana, Slovenia, we received written confirmation from the head of the school and, in the case of minors at the same school, written consent from their guardians and/or teachers. In Slovenia, the written consent of the director is considered valid for the entire institution. The following presents each evaluation step in turn.

### Experimental design: conceptualization of the pre-experiment and main experiment

Within this step, research questions, the design of the task, participants, and procedure of the main experiment were conceptualized.

#### Research questions and raised hypothesis

We identified the following research questions to be examined:

**RQ1:** What is the effect of using captions in a sign language interpreter video in terms of the level of comprehension of deaf and hard of hearing persons?

It was anticipated that the use of captions would significantly increase the level of comprehension of persons with both types of hearing loss compared to the non-use of captions.

**RQ2:** Are there statistically significant differences in the comprehension scores in all four themes (hiking, sports, culture, and shopping) between the group who was watching a sign language interpreter video with captions and the group who was watching sign language interpreter videos without captions?

The group who watched sign language interpreter videos with captions was expected to report higher comprehension scores than the group who watched the videos without captions in all four themes.

#### The task design

The task was designed in a way that participants watched four sign language interpreter videos, once with captions and the second time without captions. Each video contained 10 chunks of inter-independent information within 3–7 sentences and 40–58 words. The information was summarized in indicative sentences and content related to everyday life. Based on the chunks of information, a 10-item open-ended questionnaire was developed.

The person who interpreted in sign language was officially certified on a national level and was actively involved in the activities of the Slovenian Institute for the association of interpreters for sign language. This person also actively participated in the selection of topics for sign language interpreter videos, content preparation and questionnaires for the experiment.

In order to provide quality videos, we recorded a sign language interpreter in the studio with ‘three point lighting’. The interpreter was captured using a chroma key background from the waist up and lit by using three separate positions, so that the cameraman was able to illuminate the interpreter in any way, while also controlling (or eliminating entirely) the shading and shadows produced by direct lighting. The video was captured in high definition resolution of 1280x720 at 50 fps. In this way, a more detailed capture of finger movements was possible, which is crucial for deaf people when watching a sign language interpreter [41]. In post-production, a white background was selected. Accordingly, the caption font was white but outlined in black.

**Fig 2:**
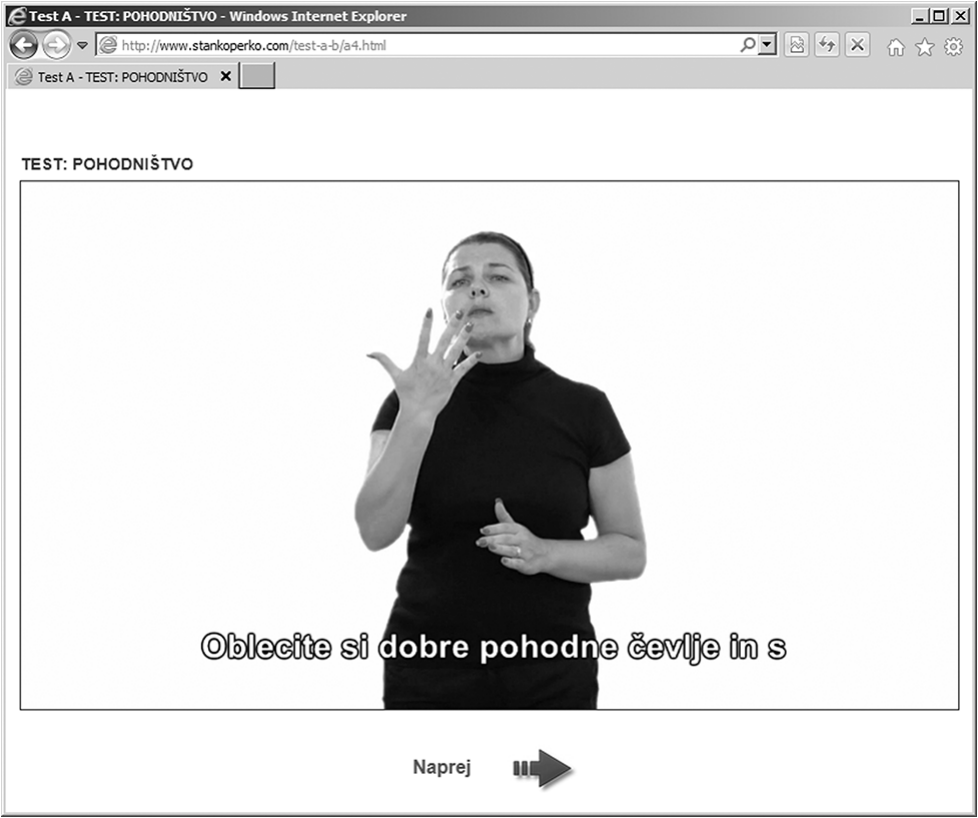
Sign language interpreter video about hiking with captions using the Sign Language Interpreter Module (in Slovene language).

For the purposes of including captions in a sign language interpreter video, we used the Sign Language Interpreter Module (SLI Module), a web-based solution for representing accessible information for deaf and hard of hearing persons (Fig 2) [42–43]. In this way, we followed the concept of including several multimedia elements, such as the video of the sign language interpreter, audio, and captions, which was substantiated in order to contribute to an increase in the users’ interest in the content of the materials while facilitating better understanding [42].

#### Participants

It was defined that a sample of Slovenian sign language users would be recruited for the main experiment. The recruitment would be randomly performed in deaf clubs across Slovenia, however, some restrictions would have to be considered. The selection of participants was proposed to be random in order to limit any possible impact on the reliability of the results. Participants were, however, required to have the ability to read and actively understand basic written text and to have basic experience in using information and communication technology. The level of written language skills was thus recognized as an issue when using captions and was considered by the authors during the selection of the sample, as performed in the study by Gulliver and Ghinea [9]. Accordingly, the addition of written language skills and experiences in using information and communication technology was considered as outside the scope of the performed experiment. However, participants’ literacy levels were roughly pre-tested by asking them to read the consent form and agree to it. Their comprehension of written text was then ascertained in a signed dialogue with a sign language interpreter, similar to the study presented by Wehrmeyer [44]. Concurrently, participants’ knowledge of sign language skills was ascertained by the sign language interpreter. Normal or corrected-to-normal vision was also a requirement. However, no restriction was made on the degree of hearing loss, age, or gender of the participants.

#### Procedure

**Fig 3:**
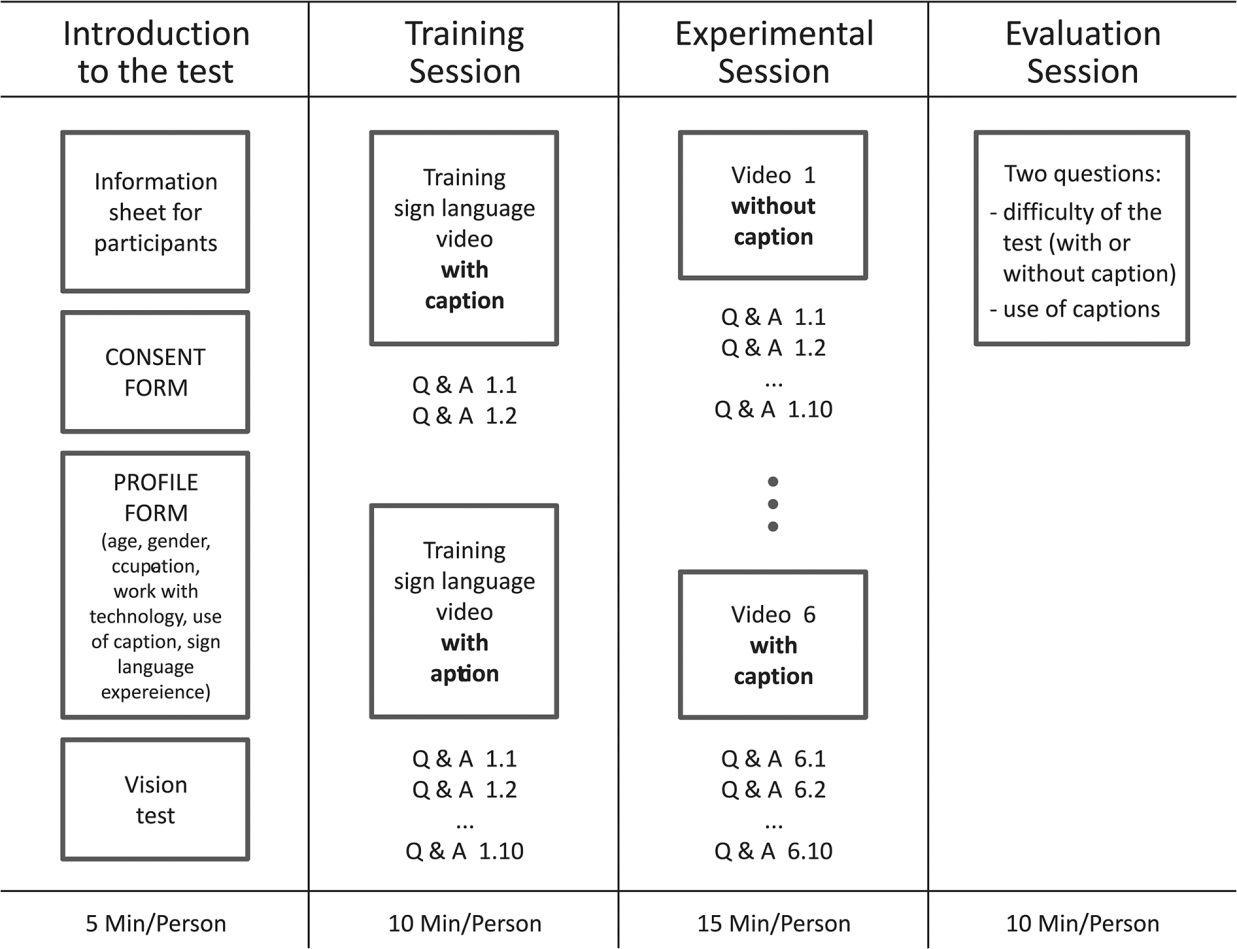
Scheme and time estimation for procedures adopted in the test.

The procedure was set to be divided into four parts (Fig 3). At first, the introduction to the test and a training session were set. The actual main experimental session and, finally, an evaluation session were proposed to follow.

*Introduction to the test*. Before starting the training session, sign language users were informed about the experiment by means of a sign language interpreter. Next, participants were provided with an information sheet and were asked to sign a written consent form. After filling out the questionnaire, where participants provided information about their age, gender, and type of hearing loss, visual tests were conducted. Participants’ binocular acuity was recorded for far (5m) and intermediate (70 cm) distances using a Landolt ring chart. Participants ran all tests with their habitual glasses in place.

*Training Session*. Two video sequences served for the training session. After the video had been displayed, participants answered questions about the information presented. The first video was short, including one statement, and participants were given just three questions. The second video was similar to the actual experimental video sequences and ten questions were provided.

*Experimental Session*. Participants watched four video sequences in total with information from everyday life, with or without captions. After the presentation of each video sequence, participants answered ten questions about the content presented.

*Evaluation Session*. After the experimental session, participants were asked to report on the subjective degree of difficulty for gathering information in sign language with or without captions. A visual analogue scale was used in order to record participants’ estimates of difficulty. It was measured with a 10-point scale with response categories ranging from 1 (very easy) to 10 (very difficult). The visual scale was plotted on a sheet of paper and participants indicated their responses by crossing the corresponding position on the scale.

After conducting these four sessions, the investigators’ assessment of the answers from the experimental session followed. Before the assessment, investigators developed and agreed upon a set of correct answers defined for each block of information, i.e. synonyms, where answers were formed in statements and individual words, or a combination thereof. Thereafter, one Slovenian investigator assessed the answers. Each correct answer in each block of information counted for one point. Finally, the points were summed up, so that the maximum possible number of points per block of information was 10.

### Pre-experiment: design of information blocks to be used in the main experiment

Within this step, our aim was to develop and identify four blocks of information to be used in the main experiment. In addition, content topics for the main experiment were defined. Initially, six blocks of information were designed for the following selected topics: weather forecast, economy, hiking, shopping, culture, sports. The topics were selected based on a sign language interpreter’s experiences and a general impression of the most frequently represented topics in the everyday life of people with hearing loss. Each block of information was comprised of 3–7 sentences with 40–58 words and 10 chunks of information. The written content was presented individually and a questionnaire was passed out in relation to the 10 chunks of information that were shown in the block. An example of a chunk of information is presented in Table 1. In particular, on the left, there are sentences for the theme hiking, and on the right there are questions for that chunk of information. Participants were required to find answers to all the questions within the 10 chunks of information.

**Table 1:** Example of block of information and a questionnaire for hiking.

Hiking:	QUESTIONS: Hiking
In three days, we will hike to a nearby hill. The trail will be 20 km long and the hill is steep. In that respect, we can expect the hike to last at least five hours. Please wear good hiking boots and bring warm clothes. Pack food rich in calories. At the final destination, we will rest for one hour. We will come home at around 10 p.m.	1. How long is the hike?
	2. When is the hike?
	3. Where are we hiking to?
	4. What kind of food should we bring?
	5. How many km is the trail?
	6. Are we expecting cold weather?
	7. What kind of shoes should we wear?
	8. When do we return home?
	9. For how long will we rest?
	10. Where is the resting spot?

The pre-experiment was performed with six Slovenian as well as with six German hearing people. This was done in order to gain relevant high quality results which could constitute the basis for future research and repetition of the main experiment in German-speaking countries.

During the pre-experiment, participants were asked to read six blocks of information in written language only and to fill out the open-ended questionnaires. The answers were assessed according to the assessment process described in the previous section. The average number of correct responses (scores) for each block, for the Slovenian version, ranged from 6 to 9. For the German version it ranged from 8 to 10. Exceptionally, in the Slovenian version, for the topic ‘sports’, and in the German version, for the topic ‘shopping’ the scores ranged from 9 to 10. Those scores were distributed normally. The averages, as well as the standard deviations of the scores, are shown in Table 2. Based on the results, the number of blocks of information was reduced from six to four for the main experiment. Accordingly, four video clips were created on the themes of hiking, shopping, culture, and sports. Two additional video clips for the themes ‘weather’ and ‘economy’, which were excluded from the main experiment, were only used as training videos before conducting the main experiment. This procedure allowed us to evaluate more effectively whether the test participants met the minimum testing requirements or not (knowledge of sign language, quality of vision and reading skills for the written word).

**Table 2:** Mean ± 1 standard score deviations (number of correctly retrieved chunks of information) for six topics presented in German and in Slovenian.

Version	Weather	Economy	Hiking	Shopping	Culture	Sports
Slovenian	5.7±3.0	7.2±2.7	7.5±1.5	7.7±1.5	7.8±2.2	8.5±2.3
German	7.8±1.2	8.0±0.9	8.7±1.0	9.7±0.5	9.2±0.4	9.3±0.5

*Notes*. Six German and six Slovenian hearing people were used for both the German and Slovenian versions, respectively. No participant had hearing loss.

### Main experiment: comprehension in sign language videos with and without captioning

#### Procedure

Within this step, we performed the main experiment, developed on the basis of experimental design and the results of the pre-experiment. In the main experiment, all four sessions were conducted: an introduction to the test, training, experimental, and evaluation session. The procedure for all sessions was described in the task design section. In what follows, we describe the experimental session procedure in more detail.

In the experimental session, each participant watched four video sequences, where one video sequence covered one topic selected in the pre-experiment. Of these, two videos contained captions and two videos did not. Accordingly, participants were divided into two groups: group A and group B. Group A watched the first video sequence containing sign language interpreting and captions, whereas group B watched the first video sequence without captions. Accordingly, the use of captions was alternated in the subsequent video sequences. The content remained in the same order at all times for all participants. After watching each video, participants were asked to answer the questionnaire.

#### Participants

Fifty-one Slovenian sign language users were included in the experiment. Table 3 shows the basic demographic characteristics. Slightly more men (58.8%) than women (41.2%) participated. On average, they were 30 years old (age range: 15–73, *SD* = 17.85) and all of them had knowledge of Slovenian sign language and Slovenian written language. Among all the participants, the largest groups were the under-20 group (32.20%) and the 21 to 40 group (37.29%). When asked to provide a self-report about their type of hearing loss, the majority of participants reported that they were deaf. For the purposes of this study, we classified participants into subgroups twice. First, they were classified into two hearing loss groups (a) deaf (*n* = 37), and (b) hard of hearing (*n* = 14). Second, they were classified into two groups regarding the sequence of playing a sign language interpreter video with or without captions: (a) group A (*n* = 25), and (b) group B (*n* = 26).

**Table 3:** Demographic data about the sample (N = 51).

Demographic data		N	%
**Participants**	Total number	51	100
	Male	30	58.8
	Female	21	41.2
**Types of hearing loss**	Deaf	37	72.5
	Hard of hearing	14	27.5
**Group**	Group A (captions in the first test video)	25	49.0
	Group B (captions in the second test video)	26	51.0

#### Statistical analyses

A two-way mixed-design between-within subjects analysis of variance (ANOVA) was conducted in order to assess the effect of caption conditions on the participants’ level of comprehension across groups with different types of hearing loss. Statistically significant differences between two independent samples were checked with a t-test [45]. All analyses were performed with SPSS version 21.0. The resulting database will be made publicly available within the Data Archiving and Networked Services (DANS).

#### Results

The first research question asked whether the use and non-use of captions in a sign language interpreter video have an impact on the level of comprehension for deaf and hard of hearing persons. In both groups, A and B, corresponding results recorded without captions were summed and named ‘Mode 1’. The results with captions were also summed and named ‘Mode 2. Table 4 shows the rounded values for the mean and standard deviation values. A two-way mixed-design ANOVA was performed to compare comprehension scores between deaf and hard of hearing users when using captions or not.

**Table 4:** Level of comprehension when using captions and with types of hearing loss.

		Deaf	Hard of hearing
**No use of captions (Mode 1)**	*Mean* $\left(\bar{x_{1}}\right)$	3.38	4.21
	*Std Dev* (*σ*_1_)	*2*.*00*	*2*.*16*
**Use of captions (Mode 2)**	*Mean* $\left(\bar{x_{2}}\right)$	4.19	6.04
	*Std Dev* (*σ*_2_)	*2*.*19*	*1*.*78*

A statistically significant interaction was found when using captions and comprehension: Wilks’ Lambda =. 89, *F*(1, 49) = 5.98, *p* <. 05, ηp^2^ =. 11. There was a significant effect for using captions: Wilks’ Lambda =. 89, *F*(1, 49) = 40.58, *p* <. 001, ηp^2^ =. 45, with both hearing loss groups showing an increase in the level of comprehension by including captions in the sign language interpreter video. It indicates that, when the captions were included in videos, comprehension scores in both groups increased. In addition, the main effect comparing the two hearing loss groups was also statistically significant, *F*(1, 49) = 4.76, *p* <. 05, ηp^2^ =. 09. It indicates that hard of hearing users, on average, reported higher comprehension scores than deaf users.

As Table 4 shows, among the deaf and hard of hearing participants, those who watched sign language interpreter videos with captions reported higher levels of comprehension than those who watched videos without captions. The increase in the level of comprehension was higher among the hard of hearing than the deaf when the sign language interpreter video contained captions. In this regard, we calculated the percentage increase in comprehension when captions were included in a sign language interpreter video compared to when they were not. The calculation was performed in accordance with the following equation:

$$\text{increment}=\frac{\bar{x_{2}}}{\bar{x_{1}}}*100-100$$ EQ.1

**Fig 4:**
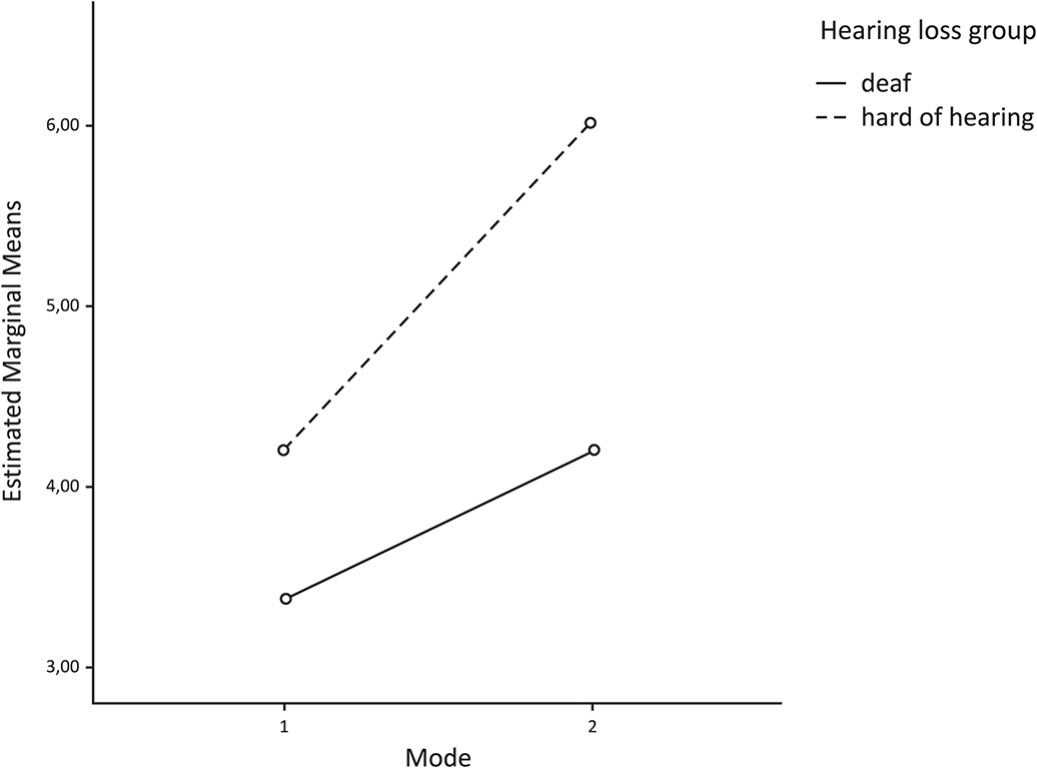
Level of comprehension when using captions and types of hearing loss.

The results showed that the comprehension of a sign language interpreter video with captions was 24% better among the deaf and 42% better among the hard of hearing (Fig 4).

The second research question sought to identify the differences between the group that watched a sign language interpreter video with captions and the group that watched the videos without captions in the comprehension scores of topics: hiking, culture, shopping, and sports. To compare the scores, a t-test was performed. Table 5 lists the numbers of correct answers (scores) for the topics. For each topic, two groups watched the same video: one group with captions and the second group without captions. Thus, the maximum score for each theme for comprehension with captions is 10, which is also the case for comprehension without captions.

**Table 5:** Comprehension scores with and without captions in the sign language interpreter video regarding the themes.

Theme	Captions	Mean	Std. Deviation	Std. Error Mean
**Hiking**	yes	5.28	2.07	.41
	no	3.69	1.85	.36
**Culture**	yes	4.60	2.90	.58
	no	3.04	2.49	.49
**Shopping**	yes	4.15	2.62	.51
	no	3.16	2.32	.46
**Sports**	yes	4.77	2.42	.48
	no	4.56	2.53	.51

The differences between the group that watched the sign language interpreter videos with captions and the group that watched them without captions were statistically significant for the hiking and culture topics, but not for shopping and sports. On average, for the hiking theme, users achieved better scores for the comprehension of sign language interpreter videos with captions, *M* = 5.28, *SD* = 2.07, *SE* =. 41, than in the comprehension of videos without captions, *M* = 3.69, *SD* = 1.85, *SE* =. 36. The difference was statistically significant, *t*(49) = 2.89, *p* <. 05. Furthermore, Cohen’s effect size (*d* =. 81) suggested a high practical significance [46].

Likewise, users reported higher comprehension scores when watching the video about culture with captions, *M* = 4.60, *SD* = 2.90, *SE* =. 58, than when captions were not included, *M* = 3.04, *SD* = 2.49, *SE* =. 49. The difference was statistically significant, *t*(49) = 2.07, *p* <. 05, and Cohen’s effect size (*d* =. 58) suggested a moderate to high practical significance [46].

The results revealed no statistically significant differences in comprehension scores when watching sign language interpreter videos with or without captions for the themes of shopping, *t*(49) = - 1.43, *p* >. 05, and sports, *t*(49) = —.30, *p* >. 05. Concerning the comparison of comprehension scores across themes, the highest score was achieved for the hiking theme and the lowest score was achieved for the shopping theme, both when captions were included and not.

## Discussion

In the present study, we aimed to compare comprehension scores for content presented in a sign language interpreter video in a combination that was with captions and without captions. The results showed that the inclusion of captions in a sign language interpreter video significantly increased the level of comprehension in both deaf and hard of hearing people. Particularly, comprehension scores improved by 24% among deaf and by 42% among hard of hearing people. In addition, hard of hearing people reported a higher level of comprehension than deaf people, in both situations, i.e. when captions were or were not included in the sign language interpreter video.

As far as the themes of videos used in the experiment were concerned, the highest differences in comprehension, when using captions or not, was evident in the sign language interpreter video for the hiking theme. The comprehension scores were significantly higher when using captions than when captions were not included. In addition, the effect of using captions was slightly lower in the sign language interpreter video for the culture theme, where comprehension scores were significantly higher when captions were included. On average, for the hiking theme, the comprehension score was the highest among all themes with the inclusion of captions. The culture theme followed. The objectivity of providing information for each video was assured by proposing the same number of pieces of information, with a similar number of sentences and words. Although all themes were from everyday life, the subjective factor could not be excluded. Thus, the differences in comprehension scores could be attributed to participants’ subjective factors. One of the reasons might be that participants have a larger set of vocabulary for the theme of hiking in Slovenian sign and/or written language.

The described findings are important as they show that the presence of captions contributes to a better comprehension of sign language interpreter videos. In addition, they can indicate that deaf and hard of hearing sign language users perceive captioning as support for signing. The findings are in line with the expected answers for both research questions, whereas the differences in comprehension scores between the use and non-use of captions in the sign language interpreter video for the shopping and sports themes were not significant.

Our findings compliment the findings of Yoon and Kim [23] and Kim and Yeong [24] who showed that captions in sign language interpreter videos positively affect deaf learners’ understanding of the content of educational material. Likewise, we substantiated a positive effect for captions with regard to the comprehension levels of deaf and hard of hearing viewers. In addition, our findings can complement the findings of Szarkowska et al. [17] who examined what type of captions in television programmes should be used for better comprehension among the deaf and hard of hearing and hearing viewers, but found no significant differences. Complementing their results, we substantiated the effects of using captions on the comprehension scores of viewers with hearing loss.

## Conclusions and Recommendations

With the present study in mind, we aimed to make a scientific contribution to the ongoing debate of whether to use captions or not in sign language interpreter videos. These findings show that the presence of captions in sign language interpreter videos improved the comprehension of deaf viewers by 24% and hard of hearing viewers by 42%. In the experiment, we used four videos, with and without captions, which briefly presented four different themes from everyday life, such as hiking, culture, shopping and sports. The first two topics showed significant differences when using or not using captions, while for the shopping and sports themes we could not confirm a significant difference, since the deviation was too high.

The present study has two main limitations. First, there are a relatively small number of deaf and hard of hearing people available in Slovenia who could be included in the sample of our study, which was also found in a study by Kožuh, Hintermair, Holzinger, Volčič, and Debevc [47]. In addition, our sample was randomly recruited from the Slovenian deaf and hard of hearing population, where 1,850 deaf and 50,000 hard of hearing people are registered [48]. In order to address the limitations related to the sample, it would be necessary to increase the sample by inviting deaf and hard of hearing people from Slovenia and other European countries to participate in the study.

The second limitation is that participants’ backgrounds were not entirely examined. In particular, we did not objectively measure written and sign language skills and we did not comprehensively address participants’ language preferences and characteristics, such as using sign language as a mother tongue or not. To address these limitations in future research, a comprehensive examination of participants’ language and education background is needed.

In addition, some issues have not been addressed yet, such as whether complexity and the difficulty of information vary across blocks of text. Important research questions that require further work are, for example, how much information is obtained from an image and how much from captions, and what the actual reading pattern is when captioned material is being viewed. Based on our results and on the results of previous studies, e.g. [12–24], we recommend establishing an international standard for using captions in sign language interpreter videos in traditional and new media. In this way we could expect even higher improvements in the comprehension rates of deaf and hard of hearing viewers.

## References

[1] Hanson VL. Computing technologies for deaf and hard of hearing In: Sears A, Jacko JA, editors. Human-Computer Interaction Handbook: Fundamentals, Evolving Technologies and Emerging Applications. 2nd ed. Hillsdale, NJ: Lawrence Erlbaum Associates; 2008; p. 885–893.

[2] Allen C, Haualand H. Deaf People and Human Rights. World Federation of the Deaf and Swedish National Association of the Deaf (Research Report) World Federation of the Deaf website. [Internet]. 2009 [cited 2014 Jul 1]. Available from: http://www.hrc.co.nz/hrc_new/hrc/cms/files/documents/06-May-2009_15-14-55_DeafPeople_HumanRightsReport.pdf

[3] Cavender AC, Ladner RE. Hearing Impairments In: Harper S, Yesilada Y, editors. Web Accessibility: A Foundation for Research. New York: Springer; 2008; p. 26–36.

[4] World Federation of the Deaf (WFD) Sign Language [Internet]. 2013 [cited 2014 Jul 1]. Available from: http://wfdeaf.org/human-rights/crpd/sign-language

[5] Marschark M, Harris M. Success and failure in learning to read: The special case of deaf children In: Cornoldi C, Oakhill J, editors. Reading comprehension difficulties: Processes and intervention. Mahwah, NJ: Lawrence Erlbaum Associates; 1996; p. 279–300.

[6] Marschark M, Sapere P, Convertino C, Seewagen R, Maltzen H. Comprehension of Sign Language Interpreting: Deciphering a Complex Task Situation. Sign Language Studies. 2004; 4(4): 345–368.

[7] Hanson VL. Progress on Website Accessibility? ACM Transactions on the Web. 2013; 7(1): Article 2.

[8] Andrew KN, Hoshooley J, Joanisse MF. Sign Language Ability in Young Deaf Signers Predicts Comprehension of Written Sentences in English. PLoS One. 2014; 9(2):e89994 doi: 10.1371/journal.pone.0089994 24587174 10.1371/journal.pone.0089994 PMC3938551

[9] Gulliver SR, Ghinea G. How Level and Type of Deafness Affects User Perception of Multimedia Video Clips. Universal Access in the Information Society. 2003a; 2(4):374–386.

[10] Gulliver SR, Ghinea G. Impact of captions on hearing impaired and hearing perception of multimedia video clips. In: Proceedings of the IEEE International Conference on Multimedia and Expo, ICME ‘02: Vol. 1. Lausanne, Switzerland: IEEE. 2003b; p. 753–756.

[11] Jelinek Lewis MS, Jackson DW. Television literacy: Comprehension of program content using closed captions for the deaf. J Deaf Stud Deaf Educ. 2001; 6(1):43–53. 15451862 10.1093/deafed/6.1.43

[12] Cambra C, Leal A, Silvestre A. How deaf and hearing adolescents comprehend a televised story. Deafness Educ Int. 2010; 12(1):34–51.

[13] Strassman BK, O’Dell K. Using open captions to revise writing in digital stories composed by d/Deaf and hard of hearing students. Am Ann Deaf. 2012; 157(4):340–357. 23259353

[14] Kirkland CE, Byrom E, MacDougall M, Corcoran M. The Effectiveness of Television Captioning on Comprehension and Preference. Annual Meeting of the American Educational Research Association [Internet]. 1995 [cited 2014 Jul 26]. Available from: http://files.eric.ed.gov/fulltext/ED389286.pdf

[15] Hong R, Wang M, Yuan X-T. Video Accessibility Enhancement for Hearing-Impaired Users. ACM Transactions on Multimedia Computing Communications and Applications. 2011; 7S(1):1–19.

[16] Cambra C, Silvestre A, Leal A. Comprehension of television messages by deaf students at various stages of education. Am Ann Deaf. 2009; 153(5):425–434. 19350951 10.1353/aad.0.0065

[17] Szarkowska A, Krejtz I, Kłyszejko Z, Wieczorek A. Verbatim, standard, or edited? Reading patterns of different captioning styles among deaf, hard of hearing, and hearing viewers. Am Ann Deaf. 2011; 156(4):363–78. 22256538 10.1353/aad.2011.0039

[18] Burnham D, Leigh G, Noble W, Jones C, Tyler M, Grebennikov L, et al Parameters in television captioning for deaf and hard-of-hearing adults: Effects of caption rate versus text reduction on comprehension. J Deaf Stud Deaf Educ 2008; 13(3):391–404. doi: 10.1093/deafed/enn003 18372297 10.1093/deafed/enn003

[19] De Linde Z, Kay N. The semiotics of subtitling Manchester, England: St. Jerome Publishing; 1999.

[20] Perego E, de Missier F, Porta M, Mosconi M. The cognitive effectiveness of subtitle processing. Media Psychology. 2010; 13:243–272.

[21] D’Ydewalle G, De Bruycker W. Eye movements of children and adults while reading television subtitles. European Psychologist. 2007; 12(3):196–205.

[22] D’Ydewalle G, Rensbergen JV, Pollet J. Reading a message when the same message is available auditorily in another language: The case of subtitling In: JK O’Reagan JK, Lévy-Schoen A, editors. Eye movements: From physiology to cognition. Amsterdam, Netherlands: Elsevier; 1987, p. 313–321.

[23] Yoon JO, Kim M. The effects of captions on deaf students’ content comprehension, cognitive load, and motivation in online learning. Am Ann Deaf. 2011; 156(3):283–289. 21941878 10.1353/aad.2011.0026

[24] Kim Y, Yeong E. The effect of a caption video program on the written skills of students with hearing impairment. The Journal of Special Education: Theory and Practice. 2006; 7(1):489–506.

[25] Deaf News Network (DNN). FCC Requires Captioning on Video Clips from TV Starting in 2016 [Internet]. 2014 [cited 2014 Jul 26]. Available from: http://deafnews.net/index.php/politics/nad/item/304-fcc-requires-captioning-on-video-clips-from-tv-starting-in-2016

[26] European Federation of Hard of Hearing People. State of subtitling access in EU 2011 [Internet] 2011 [cited 2015 Feb 27]. Available from: http://ec.europa.eu/internal_market/consultations/2011/audiovisual/non-registered-organisations/european-federation-of-hard-of-hearing-people-efhoh-_en.pdf

[27] European Commission. European disability strategy 2010–2020—Initial plan to implement the European disability strategy 2010–2020—list of actions 2010–2015 [Internet]. 2010 [cited 2015 Feb 27]. Available from: http://eur-lex.europa.eu/LexUriServ/LexUriServ.do?uri=SEC:2010:1324:FIN:EN:PDF

[28] European Commission. European disability strategy 2010–2020—a renewed commitment to a barrier-free Europe [Internet]. 2010 [cited 2015 Feb 27]. Available from: http://eur-lex.europa.eu/LexUriServ/LexUriServ.do?uri=COM:2010:0636:FIN:EN:PDF

[29] UN General Assembly. Convention on the Rights of Persons with Disabilities: Resolution / adopted by the General Assembly, A/RES/61/106 [Internet]. 2007 [cited 2015 Feb 27]. Available from: http://www.refworld.org/docid/45f973632.html

[30] Schirmer BR. Dimensions of deafness: Psychological, social and educational Boston: Allyn & Bacon; 2001.

[31] Fajardo I, Cañas J, Salmerón L, Abascal J. Towards a cognitive accessibility guideline based on empirical evidences of deaf users web interaction. In: HCI International. London, UK: Lawrence Erlbaum Associates. 2003; p. 950–954.

[32] Koskinen PS, Wilson RM, Gambrell L, Neuman SB. Captioned video and vocabulary learning: Innovative practices in literacy instruction. Read Teach. 1993; 47:36–43.

[33] Sadler KL. Accuracy of Sign Interpreting and Real-Time Captioning of Science Videos for the Delivery of Instruction to Deaf Students [Internet]. Doctoral dissertation, University of Pittsburgh, USA. 2009. Available from: http://d-scholarship.pitt.edu/8488/1/SadlerKarenL20509.pdf

[34] Paivio A. Mental representations: a dual coding approach Oxford. England: Oxford University Press; 1986.

[35] Brickman B, Workman S. Enhancing the learning environment for deaf students in the Science Classroom. Journal of Science for Persons with Disabilities. 1995; 3:40–43.

[36] Nugent G. Deaf students’ learning from captioned instruction: The relationship between the visual and caption display. The Journal of Special Education. 1983; 17:227–234.

[37] Steinfield A. The benefit of real-time captioning in a mainstream classroom as measured by working memory. Volta Review. 1998; 100:29–44.

[38] Margaret SJ, Dorothy WJ. Television literacy: Comprehension of program content using closed caption for the deaf. J Deaf Stud Deaf Educ. 2001; 6(1):43–53. 15451862 10.1093/deafed/6.1.43

[39] Chisholm W, Vanderheiden G, Jacobs I. Web content accessibility guidelines 1.0. Interactions. 2001; 8(4):35–54.

[40] European Broadcasting Union (EBU). Access Services includes recommendations (Research Report). European Broadcasting Union website [Internet]. 2004 [cited 2014 Jun 20]. Available from: http://www.ebu.ch/CMSimages/fr/tec_text_i44-2004_tcm7-14894.pdf

[41] Debevc M, Kosec P, Holzinger A. E-learning accessibility for the deaf and hard of hearing—practical examples and experiences. Lecture Notes in Computer Science. 2010; 6389:203–213.

[42] Debevc M, Kosec P, Holzinger A. Improving multimodal web accessibility for deaf people: sign language interpreter module. Multimed Tools Appl. 2011; 54(1):181–199.

[43] Holzinger A. Usability Engineering for Software Developers. Commun ACM. 2005; 48:71–74.

[44] Wehrmeyer J. Eye-tracking Deaf and hearing viewing of sign language interpreted news broadcasts Journal of Eye Movement Research. 2014; 7(1):3:1–16.

[45] Howell D. Statistical Methods for Psychology. Pacific Grove, CA: Duxbury; 2002.

[46] Cohen J. Statistical Power Analysis for the Behavioral Sciences, 2nd Ed. Hillsdale, NJ: Lawrence Earlbaum Associates; 1988.

[47] Kožuh I, Hintermair M, Holzinger A, Volčič Z, Debevc M. Enhancing Universal Access: Deaf and Hard of Hearing People on Social Networking Sites. Universal Access in the Information Society. 2014; 1–9.

[48] European Union of the Deaf. Slovenia [Internet]. 2012 [cited 2014 Jun 23]. Available from: http://www.eud.eu/Slovenia-i-200.html

